# Disease-associated mutations in human *TUBB3* disturb netrin repulsive signaling

**DOI:** 10.1371/journal.pone.0218811

**Published:** 2019-06-21

**Authors:** Qiangqiang Shao, Tao Yang, Huai Huang, Tanushree Majumder, Bhakti Ajit Khot, Mohammad Masoudian Khouzani, Farrah Alarmanazi, Yasmin K. Gore, Guofa Liu

**Affiliations:** Department of Biological Sciences, University of Toledo, Toledo, OH, United States of America; University of Queensland, AUSTRALIA

## Abstract

Missense mutations in the human *TUBB3* gene cause a variety of neurological disorders associated with defects in axon guidance and neuronal migration, but the underlying molecular mechanisms are not well understood. Recent studies have shown that direct coupling of dynamic TUBB3 in microtubules with netrin receptors is required for netrin-1-mediated axon guidance, and the interaction of netrin-1 repulsive receptor UNC5C with TUBB3 is involved in netrin-1 mediated axonal repulsion. Here, we report that *TUBB3* mutations perturb netrin-1/UNC5C repulsive signaling in the developing nervous system. Among twelve mutants reported in previous studies, five of them show significantly reduced interaction with UNC5C in comparison to the wild-type TUBB3. TUBB3 mutants R262C and R62Q exhibit decreased subcellular colocalization with UNC5C in the peripheral area of the growth cone of primary mouse neurons. Netrin-1 reduces the colocalization of UNC5C with wild-type TUBB3, but not TUBB3 mutants R262C or R62Q, in the growth cone. Results from the *in vitro* cosedimentation assay indicate that netrin-1 inhibits cosedimentation of UNC5C with polymerized microtubules in primary mouse neurons expressing the wild-type TUBB3, but not R262C or R62Q. Expression of either R262C or R62Q not only blocks netrin-1-induced growth cone collapse and axonal repulsion of primary EGL cells *in vitro*, but also results in axon projections defects of chicken dorsal root ganglion neurons *in ovo*. Our study reveals that human *TUBB3* mutations specifically perturb netrin-1/UNC5C-mediated repulsion.

## Introduction

Microtubules are polarized hollow structure assembled by guanosine triphosphate (GTP)-dependent polymerization of α/β tubulin heterodimers. Dynamic microtubules in the axonal growth cone (GC) of developing neurons could function as a direct sensor to control axon extension and pathfinding [1–14]. Mutations in human α- and β-tubulin encoding genes are implicated in a wide spectrum of brain malformations, such as lissencephaly, polymicrogyria, schizencephaly, hypoplasia or agenesis of the midline commissural structures (anterior commissure, corpus callosum, and fornix), hypoplasia of the internal capsule, the corticospinal tract, the oculomotor and optic nerves, dysmorphisms of the basal ganglia, the hippocampus, cerebella, and brainstem [6, 15–22]. There is a growing body of evidence indicating that the spectrum of tubulin mutation-related tubulinopathies is associated with specific defects in neuronal migration and axonal guidance [13, 16, 17, 23–25]. β-tubulin III (TUBB3), a highly dynamic tubulin isotype, is predominantly expressed in developing neurons [13, 23–26]. Recent genetic and functional studies have shown that missense mutations in the *TUBB3* gene disturb microtubule dynamics, impair kinesin interactions, and cause various neurological disorders characterized by defects in axon guidance and neuronal migration that include agenesis or hypoplasia of anterior commissure (AC), corpus callosum (CC), corticospinal tracts, and cranial nerves as well as malformations of cortical development (MCD) associated with neuronal migration and differentiation abnormalities [16, 17]. These findings demonstrate that TUBB3 is specifically involved in axon guidance during brain development, but the underlying molecular mechanisms are not well characterized.

It is believed that coupling signal transduction cascades downstream of guidance receptors to microtubule dynamics is a key event for neurons to maneuver GC navigation [1, 2, 4, 6, 9]. However, recent studies suggest a model that direct interaction of guidance receptors with microtubules via TUBB3 modulates microtubule dynamics in the GC to mediate guidance-dependent axon projection and pathfinding [13, 23–25]. For instance, netrin-1, a classic bifunctional guidance cue, is capable of attracting or repelling axon projection by differentially interacting with its receptors, deleted in colorectal cancer (DCC) [27, 28], neogenin [28, 29], uncoordinated-5 (UNC5) [30, 31], and Down syndrome cell adhesion molecule (DSCAM) [32, 33]. Netrin-1 can regulate microtubule dynamics in the GC through the direct interaction of DCC and DSCAM with TUBB3 to mediate netrin-1-induced axon outgrowth, branching, and attraction [23, 25]. Heterozygous missense mutations in the human *TUBB3* gene specifically inhibit the interaction with DCC and perturb netrin-1 attraction [24]. Interestingly, uncoupling of netrin repulsive receptor UNC5C with polymerized TUBB3 in microtubules is involved in netrin-1-mediated axon repulsion [13]. The phenotypic defects caused by *TUBB3* mutations in corticospinal tract pathfinding, trochlear axon guidance, and interhemispherical commissural axon projection in a subset of callosal neurons [16, 17] are similar to those in netrin-1^-/-^ and UNC5C^-/-^ mouse embryos [34–38]. These data suggest that *TUBB3* mutations could specifically disturb netrin-1/UNC5C signaling, resulting in netrin-1-mediated repulsion defects. In this study, we investigated the role of *TUBB3* mutations in netrin-1/UNC5C repulsion through *in vitro* and *in vivo* approaches. Our results indicate that *TUBB3* mutations could disrupt the interaction of UNC5C with polymerized TUBB3 in microtubules, resulting in defects in netrin-1-mediated axonal repulsion in the developing nervous system.

## Materials and methods

This study was carried out in strict accordance with the recommendations in the Guide for the Care and Use of Laboratory Animals of the National Institutes of Health and specifically approved by the Institutional Animal Care and Use Committee (IACUC) of the University of Toledo.

**Materials:** We used the following antibodies: rabbit anti-FLAG (Abcam catalog #ab124462, RRID:AB_11000959), rabbit anti-UNC5C (Abcam catalog #ab89938, RRID:AB_2050439), rabbit anti-hemagglutinin (HA) (Santa Cruz Biotechnology catalog #sc-805, RRID:AB_631618), mouse anti-TUBB3 (Covance catalog #MMS-435P, RRID:AB_2313773), mouse BEN antibody (DSHB, RRID:AB_2313998), bovine anti-mouse IgG-HRP (Santa Cruz Biotechnology catalog #sc-2371, RRID:AB_634824), goat anti-rabbit IgG-HRP (Santa Cruz Biotechnology catalog #sc-2004, RRID:AB_631746), AlexaFluor-488 goat anti-mouse IgG (Invitrogen catalog #A-21121, RRID:AB_141514), and AlexaFluor-647 goat anti-rabbit IgG (Invitrogen catalog #A-21244, RRID:AB_141663).

Plasmids encoding the full-length human TUBB3, UNC5C and TUBB3 mutants (A302T, R262C, R62Q, M323V, E410K, D417H, R262H, G82R, T178M, E205K, M388V, and R380C) have been described previously [13, 16, 24]. Control shRNA and TUBB3 shRNA targeting the 3’ untranslated region (UTR) of human and mouse *TUBB3* were validated in a previous study [24].

Netrin-1 was either obtained from R&D Systems or purified with anti-Myc tag affinity matrix from the conditioned media of HEK cells stably secreting Myc-tagged netrin-1. The control was made by sham purification from the conditioned media from control HEK cells. Alexa Fluor 555 phalloidin and DAPI were purchased from Invitrogen (Carlsbad, CA, USA). Taxol and nocodazole were from MP Biochemicals (Solon, OH, USA).

### Primary cerebellar neuron cultures

Cerebellar neurons were prepared from postnatal mouse pups of either sex euthanized by decapitation using scissors or cervical dislocation. The procedures of primary neuron dissociation, nucleofection and culture were as described previously with some modifications [13, 39]. Dissociated neurons from postnatal day 4 (P4) mouse cerebella or P2 external granule layer (EGL) were nucleofected (Amaxa) using program G-013. Transfected P4 cerebellar neurons were cultured on poly-L-lysine (PLL, 200 μg/ml)-coated Petri dishes overnight before immunoblotting analysis. For the Dunn chamber axon guidance assay and immunofluorescence, P2 cerebellar EGL neurons after nucleofection were grown on PLL-coated coverslips (Erie Scientific) and cultured in DMEM/B27 culture media (DMEM + B27 + 20 U/ml of penicillin/streptomycin) at 37°C with 5% CO2 for 2–3 d.

### Immunoprecipitation and immunoblotting

HeLa cells were transfected with the PEI method and cultured as previously described [13, 24]. Transfected HeLa cells and primary neurons were cultured in serum-free DMEM media for 6 h followed by incubation with either control or netrin-1 media for 5 min. The netrin-1 media was either netrin-1-conditioned media or DMEM/B27 culture media with purified netrin-1 protein (200 ng/ml). The control media were made by either conditioned media from regular HEK293 cells or sham purification from the conditioned media of HEK293 cells. Cell lysates from HeLa cells and primary neurons were immunoprecipitated with specific antibodies and protein A/G-agarose beads (Santa Cruz Biotechnology) overnight at 4°C, as described previously [13, 24]. For western blot analysis, protein extracts were separated with 7.5% SDS-PAGE and immunoblotted with designed antibodies. Western blots were visualized with the enhanced chemiluminescence kit (Fisher, Pittsburgh, PA).

### Immunocytochemistry

After nucleofection, primary EGL neurons from P2 mouse cerebella were plated on PLL-coated coverslips and cultured with DMEM/B27 culture media overnight. Primary neurons were stimulated with either sham purified control or purified netrin-1 (200 ng/ml) for 5min, followed by fixation with pre-warmed 4% paraformaldehyde (PFA) in DMEM at 37°C for 30 min. Cells were permeabilized with PBST (0.5% Triton X-100 in PBS) for 15 min, blocked with 0.25% BSA + 0.1% Triton in PBS at room temperature for 30 min, and then incubated with primary antibody solution containing rabbit anti-UNC5C and mouse anti-FLAG antibodies overnight at 4°C. After incubation with fluorescent secondary antibodies (AlexaFluor-488 donkey anti-mouse IgG and AlexaFluor-647 donkey anti-rabbit IgG), coverslips with neurons were mounted onto glass slides. Sequential fluorescent images of axonal GCs were taken using a confocal microscope with a HyD detector (Leica Microsystems, TCS-SP8) in photon counting mode. The Pearson correlation coefficient (PCC) in the peripheral region of GCs, including lamellipodia and filopodia, was obtained using both Fiji (RRID: SCR_002285) and the colocalization module of Leica Microsystems confocal software (RRID: SCR_013673). A one-way ANOVA with Tukey's test for post hoc comparisons was used to assess the significant difference of PCC values in different groups.

### Microtubule cosedimentation assay

Primary cerebellar neurons from P4 mice were dissociated, nucleofected, cultured and stimulated with purified netrin-1 (200 ng/ml) or sham-purified control for 5 min as previously described [13, 39]. Neurons were lysed in the RIPA buffer (25 mM Tris, 150 mM NaCl, 0.1% SDS, 5 mM EDTA, 0.5% sodium deoxycholate, 1% NP-40, pH 8) mixed with Roche protease inhibitor cocktail tablets. Cell lysates were centrifuged at 100,000 × g for 1 h at 4°C, and the supernatant was then incubated with 40 μM Taxol or DMSO in PEMG buffer (100 mM PIPES, 1 mM EGTA, 1 mM MgSO4, 1 mM GTP, pH 6.8) at room temperature for 30 min to stabilize microtubules. Microtubules were pelleted by centrifugation through a 10% sucrose cushion at 50,000 × g for 30 min at 25°C, and the pellet was then re-suspended in tubulin buffer (50 mM HEPES, 1 mM MgCl2, 1 mM EGTA, 10% glycerol, 150 mM KCl, 40 mM taxol, 1 mM GTP, 5 mM Mg-ATP, 1 mM PMSF, 1 x protease inhibitor mixture) as previously described [13, 24, 25]. Proteins in the supernatant and the pellet were detected by Western blotting.

### Dunn chamber axon guidance assay

The procedures of primary P2 cerebellar EGL neuron dissociation, nucleofection, and the Dunn chamber assembly were performed as previously described [13, 40] with some modification. The inner and outer wells of the Dunn chamber were filled with DMEM/B27 culture media before a coverslip with neurons was inverted over the chamber. Three sides of the coverslip were sealed with Petroleum Jelly (VL-JON Laboratory), but leaving a narrow slit at the edge of one side. After media in the outer well was drained and replaced by DMEM/B27 culture media containing either sham-purified control or purified netrin-1 (200 ng/ml), the narrow slit was sealed with Petroleum Jelly. Live cell imaging was acquired every 5 min for 90 min using a confocal microscope (Leica Microsystems, TCS SP8) to track GC navigation of YFP-labeled EGL neurons in the bridge region of the Dunn chamber. Most GCs of EGL cells showed a significant response to netrin-1 repulsion within 40–55 min, referring as ‘the turning stage’. Axon turning (angle turned) was calculated by the angle between the initial and final angel of an axon as described previously [13, 40].

### GC collapse assay

Primary EGL neurons from P2 mouse cerebella were dissociated, nucleofected, and cultured as previously described [13, 41]. Transfected neurons were treated with either sham purified control or purified netrin-1 (200 ng/ml) for 30 min. After fixed with 4% PFA for 15 min, neurons were stained with Alexa Fluor 555 phalloidin and DAPI (Invitrogen, CA, US). Images of only YFP-labeled cells were taken using a confocal microscope (Leica Microsystems, TCS SP8). The GC collapse phenotype was defined if the GC of a clearly polarized axon had no lamellipodia and two or fewer filopodia [41, 42]. At least 150 random GCs were measured per group and experiments were done in triplicate. A one-way ANOVA with Tukey's test for post hoc comparisons was performed to detect statistical significance among different groups.

### Axon projection of chicken dorsal root ganglion (DRG) neurons *in vivo*

Fertilized White Leghorn chicken eggs were incubated, and embryonic development was determined by the Hamburger and Hamilton (HH) staging series [43]. At HH stages 12–16, Venus-YFP plus specific plasmids were injected into the neural tube of chicken embryos, and the *in ovo* electroporation was performed as described previously [13]. Lumbosacral segments of the chick spinal cords labeled with YFP fluorescence were collected from chicken embryos at HH stages 23–25, and transverse 200 μm sections of the spinal cord were prepared. After fixed in 4% PFA in 1 × PBS, spinal cord slices were permeabilized with PBST (1% Triton X-100 in 1 × PBS) and then stained with BEN antibody (SC1/DM-GRASP protein). The fluorescence images of DRG axons were obtained under a confocal microscope (Leica Microsystems, TCS, SP8), and the size of the dorsal root entry zone (DREZ) was measured using the Leica Microsystems Imaging software (Leica Microsystems, Application Suite X) as described previously [13]. Data were analyzed with a one-way ANOVA followed by Tukey's test for post hoc comparisons.

## Results

### *TUBB3* mutations disrupt the interaction of UNC5C with TUBB3

**Fig 1:**
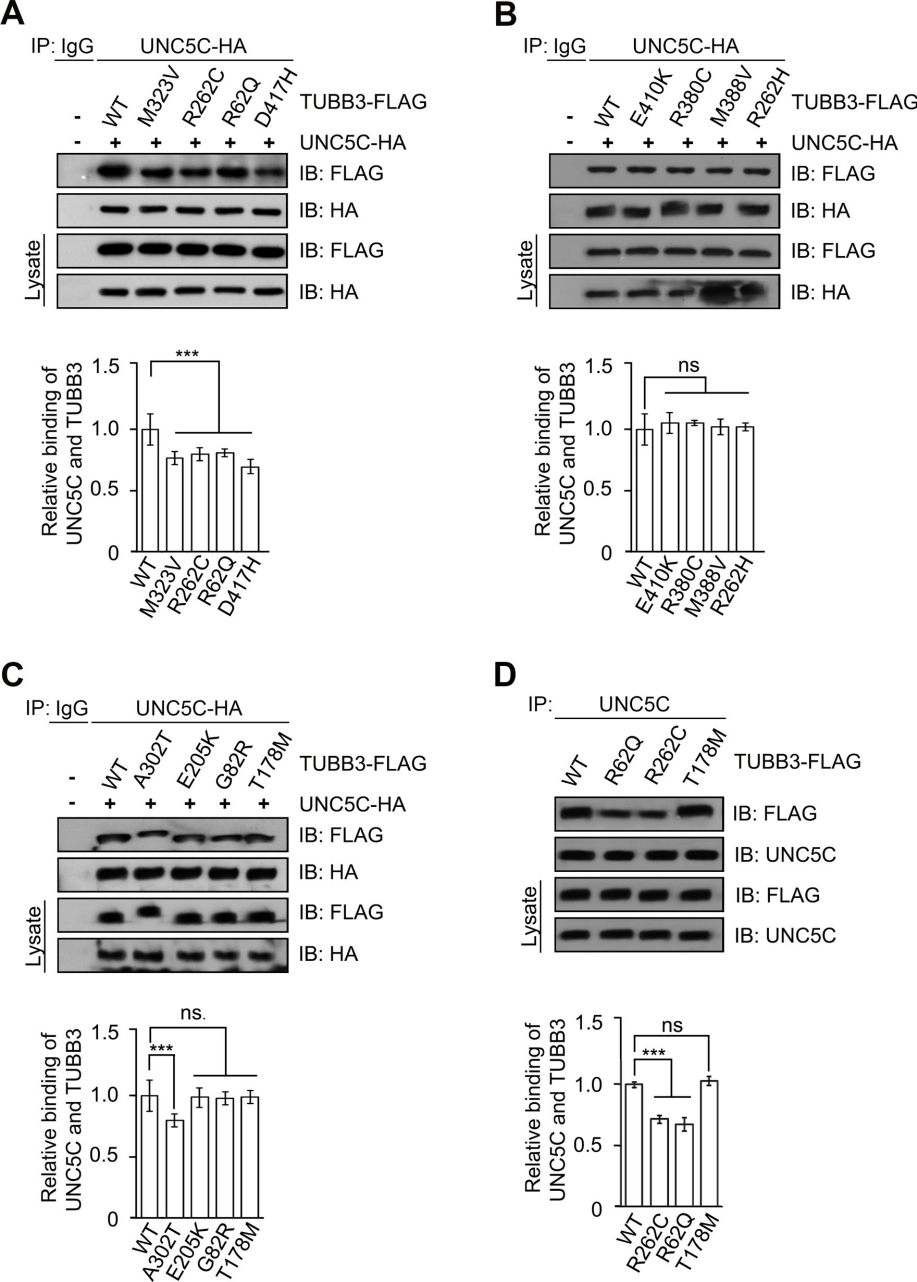
Interaction of UNC5C with TUBB3 mutants. A-C, HeLa cells were transfected with human full-length UNC5C-HA plus wild-type human TUBB3-FLAG (A–C) or FLAG-tagged TUBB3 mutants (M323V, R262C, R62Q, and D417H in A, E410K, R380C, M388V, and R262H in B, A302T, E205K, G82R, and T178M in C). The anti-HA antibody was used to immunoprecipitate UNC5C-HA and the blot analyzed with anti-FLAG and anti-HA antibodies. Relative binding of UNC5C to wild-type TUBB3 or TUBB3 mutants in A, B and C were quantified in the corresponding lower panel. D, P4 mouse cerebellar neurons were nucleofected with TUBB3 shRNAs plus either wild-type TUBB3 or TUBB3 mutants R262C, R62Q and T178M. Cell lysates of primary neurons were immunoprecipitated with anti-UNC5C and followed by probing with anti-FLAG and anti-UNC5C. Relative binding of UNC5C to TUBB3-FLAG was quantified in the lower panel. The Y axis is the normalized ratio of mean intensity (arbitrary units) of TUBB3-FLAG to HA in A-C or to endogenous UNC5C in D. Data are mean±SEM from three separate experiments, in which distinct samples were collected from three independent transfection. ns, not significant; ***, *p*<0.001 (one-way ANOVA and Tukey’s test for post hoc comparisons).

**Fig 2:**
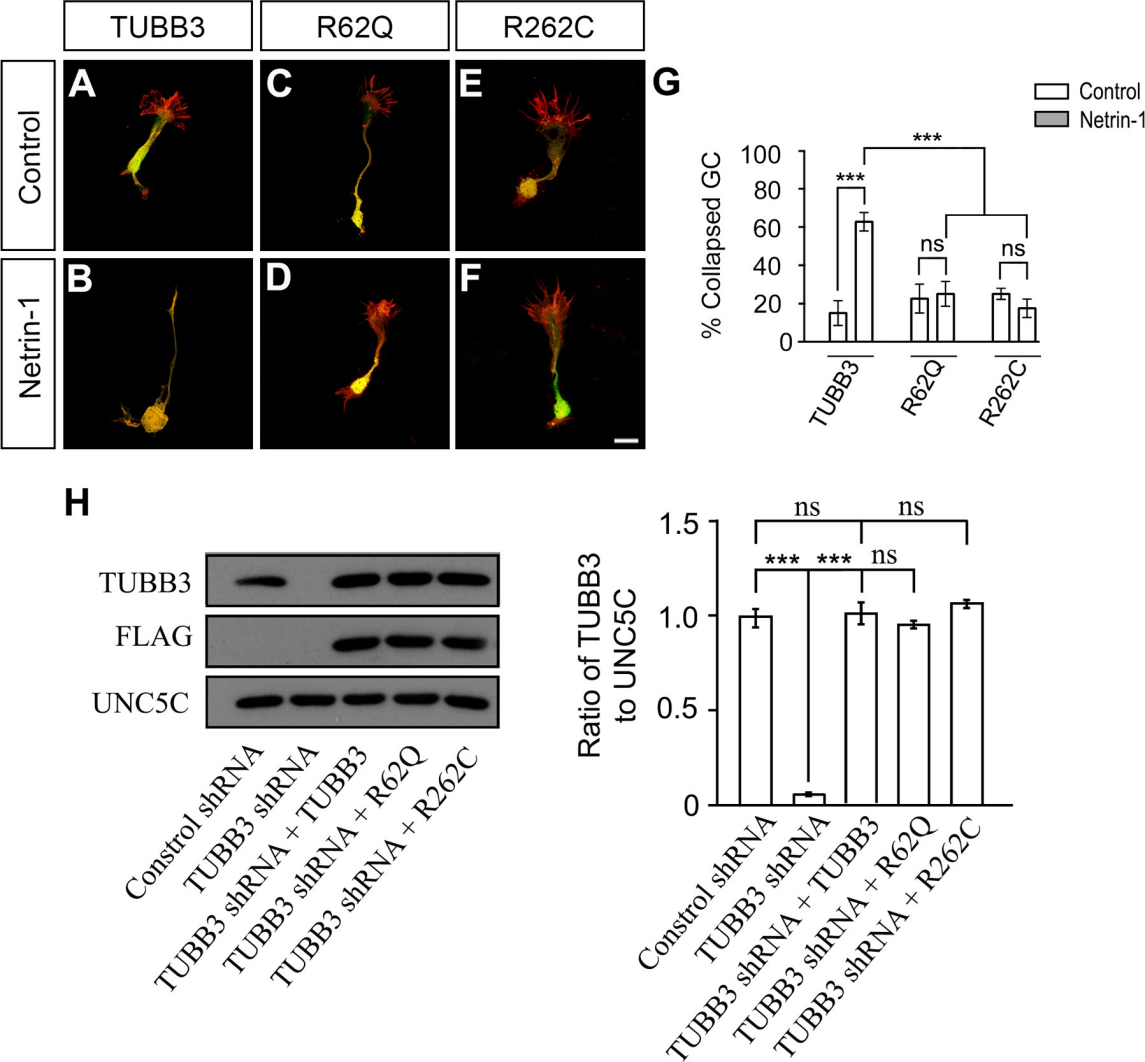
*TUBB3* mutations disturb netrin-1-induced GC collapse of EGL neurons. P2 mouse cerebellar EGL cells were nucleofected with TUBB3 shRNAs plus either wild-type TUBB3 (A and B) or TUBB3 mutants (R62Q in C and D and R262C in E and F). Neurons were stimulated with either sham purified control (A, C, and E) or purified netrin-1 (B, D, and F). Primary neurons were stained with Alexa Fluor 555 phalloidin (red) and DAPI (blue). Venus-YFP-transfected neurons (green) were imaged using a confocal microscope. Only the GCs of YFP-positive neurons not in contact with other EGL cells were measured and used in the statistical analyses. Scale bar, 10 μm. G, Quantification of the percentage of collapsed axonal GCs. In total, more than 150 neurons/group from three independent experiments were analyzed. Data are mean±SEM. ns, not significant; ***, *p* < 0.001 (one-way ANOVA and Tukey’s test for post hoc comparisons). H, Primary neurons from P4 mouse cerebella were transfected with control shRNA, TUBB shRNAs, TUBB3 shRNAs plus wild-type human TUBB3, TUBB3 shRNAs plus R262C, and TUBB3 shRNAs plus R62Q. Expression of TUBB3 and UNC5C was examined by Western blotting. The right panel is the quantification of TUBB3 and UNC5C levels from three independent experiments. Data are mean±SEM. ns, not significant; ***, p < 0.001 (one-way ANOVA and Tukey’s test for post hoc comparisons).

The direct interaction of UNC5C with TUBB3 is involved in netrin-1/UNC5C-dependent axon repulsion [13]. To examine the effect of missense *TUBB3* mutations on the interaction with UNC5C, we co-transfected plasmids encoding full-length human UNC5C tagged with human influenza hemagglutinin (UNC5C-HA) with either full-length FLAG-tagged human TUBB3 (TUBB3-FLAG) or TUBB3 mutants (M323V, R262C, R62Q, D417H, E410K, R380C, M388V, R262H, A302T, E205K, G82R, and T178M) into HeLa cells. The results of co-immunoprecipitation (co-IP) showed that the interaction of UNC5C with five of twelve TUBB3 mutants (A302T, M323V, R262C, R62Q, and D417H) was significantly reduced, compared to the wild-type TUBB3 (Fig 1A–1C, quantification in lower panels). Endogenous UNC5C interacts with TUBB3 in primary cerebellar neurons from postnatal day 4 (P4) mice [13]. To determine whether missense *TUBB3* mutations affect the interaction with endogenous UNC5C, we transfected TUBB mutants R262C, R62Q, and T178M into P4 mouse cerebellar neurons and performed a co-IP experiment (Fig 1D). R262C and R62Q are the most and least frequent mutant substitutions associated with defects in microtubule incorporation and axon guidance, respectively [17]. T178M was transfected as a control because it did not disturb the interaction with UNC5C in HeLa cells (Fig 1C). To minimize the interference of endogenous TUBB3 on the interaction with UNC5C, the previously validated TUBB3 shRNA targeting 3’UTR of human and mouse *TUBB3* [24] was co-transfected with either wild-type TUBB3 or designed TUBB3 mutants (R262C, R62Q, and T178M) into primary neurons (Figs 1D and 2H). TUBB3 shRNAs knocked down endogenous TUBB3 and transfection of exogenous FLAG-tagged TUBB3, R262C, and R62Q into primary neurons after TUBB3 knockdown could rescue TUBB3 expression at equivalent levels to endogenous TUBB3 (Fig 2H). Expression of R262C and R62Q, but not T178M, reduced the interaction with endogenous UNC5C, compared to the wild-type TUBB3 (Fig 1D). These results indicate that *TUBB3* mutation could differentially impair the interaction with UNC5C.

### *TUBB3* mutations perturb netrin-1-mediated interaction of UNC5C with polymerized TUBB3 in microtubules

**Fig 3:**
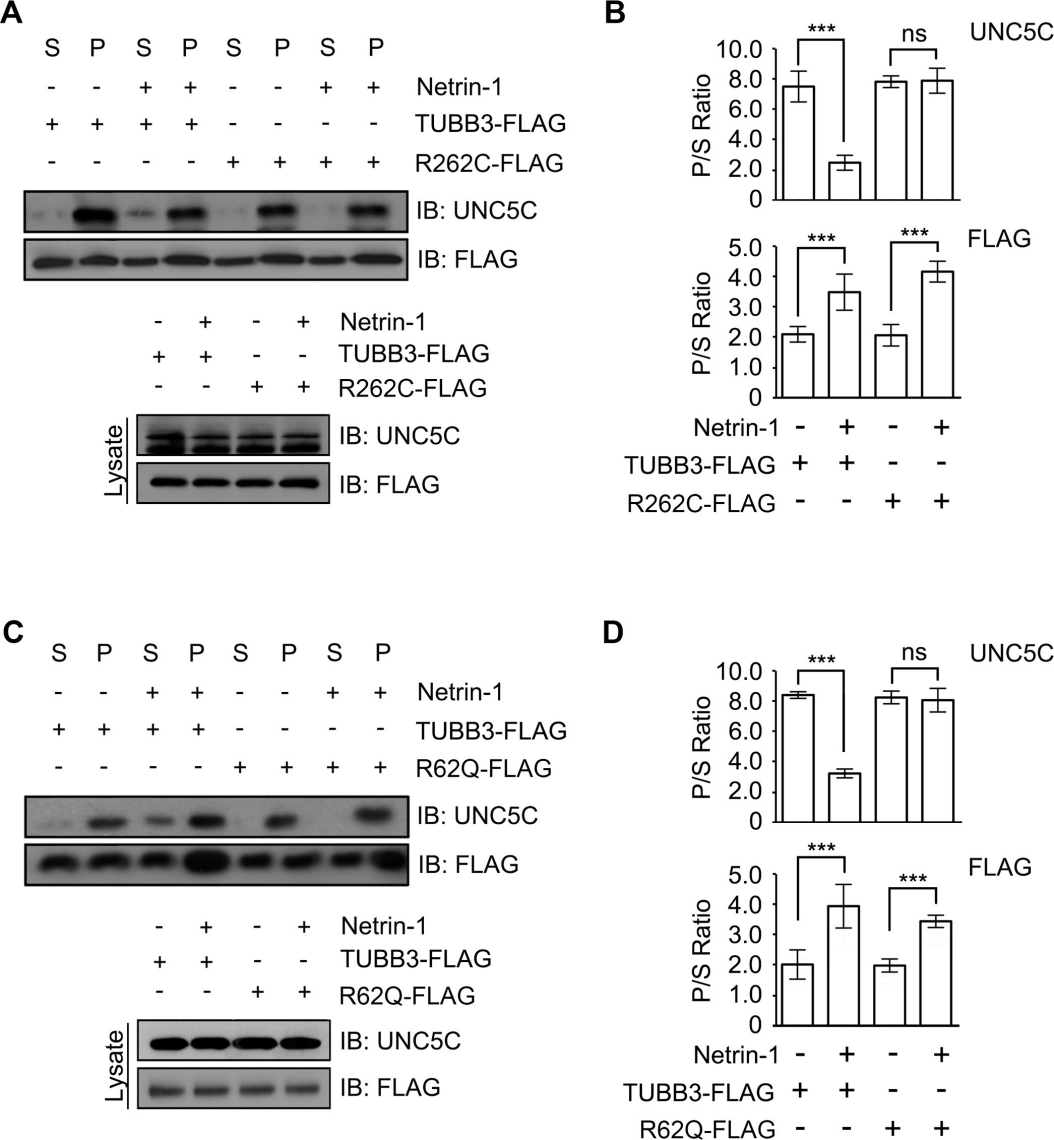
*TUBB3* mutations disrupt the netrin-1-reduced interaction of UNC5C with polymerized TUBB3 in microtubules. Primary P4 mouse cerebellar neurons were nucleofected with TUBB3 shRNAs plus either wild-type TUBB3 (A-D) or TUBB3 mutants (R262C in A-B and R62Q in C-D) prior to stimulating with netrin-1 or sham-purified control, and a cosedimentation assay of cell lysates was performed. UNC5C and wild-type TUBB3 or its mutant variants in the pellet (P) and supernatant (S) fractions were examined by immunoblotting using anti-UNC5C and anti-FLAG antibodies, respectively. B and D are the quantification of A and C from three independent experiments, respectively. Data are mean±SEM. ns, not significant; ***, *p* < 0.001 (one-way ANOVA and Tukey’s test for post hoc comparisons).

Apart from the direct association between UNC5C and unpolymerized TUBB3, the interaction of UNC5C with dynamic (polymerized) TUBB3 in microtubules is required for netrin-1-mediated axonal repulsion [13]. To determine whether *TUBB3* mutations affect the interaction of endogenous UNC5C with polymerized TUBB3, TUBB3 shRNAs were nucleofected with wild-type human TUBB3-FLAG, R262C-FLAG or R62Q-FLAG into P4 mouse cerebellar neurons. After transfection, primary neurons were then cultured and stimulated with either purified netrin-1 or sham-purified control for 5 min. Neuron lysates were incubated with taxol to stabilize microtubules *in vitro* at room temperature followed by a microtubule cosedimentation assay as previously described [13, 24]. Unpolymerized TUBB3 in the soluble supernatant (S) and polymerized TUBB3 in the pellet (P) were separated using ultra-centrifugation. The ratio of TUBB3 in P to S (P/S Ratio) showed a significant increase after netrin-1 stimulation in the wild-type TUBB3, R262C and R62Q groups (Fig 3A–3D), suggesting that these mutants do not interfere netrin-1-promoted microtubule polymerization in P4 cerebellar neurons. Netrin-1 stimulation could decrease P/S ratio of wild-type UNC5C, indicating that netrin-1 reduces the interaction of endogenous UNC5C with polymerized TUBB3 in microtubules, which is consistent with the previous study [13]. However, expression of either R62Q or R262C blocked the netrin-1 inhibition on the P/S ratio of UNC5C with more UNC5C remained in the P than in the S compared to the wild-type TUBB3 group (Fig 3A–3D). These data suggest that both R262C and R62Q mutants impair netrin-1-regulated interaction of UNC5C with polymerized TUBB3 in microtubules.

### *TUBB3* mutations disturb the colocalization of UNC5C with TUBB3 in the GC of primary EGL cells

**Fig 4:**
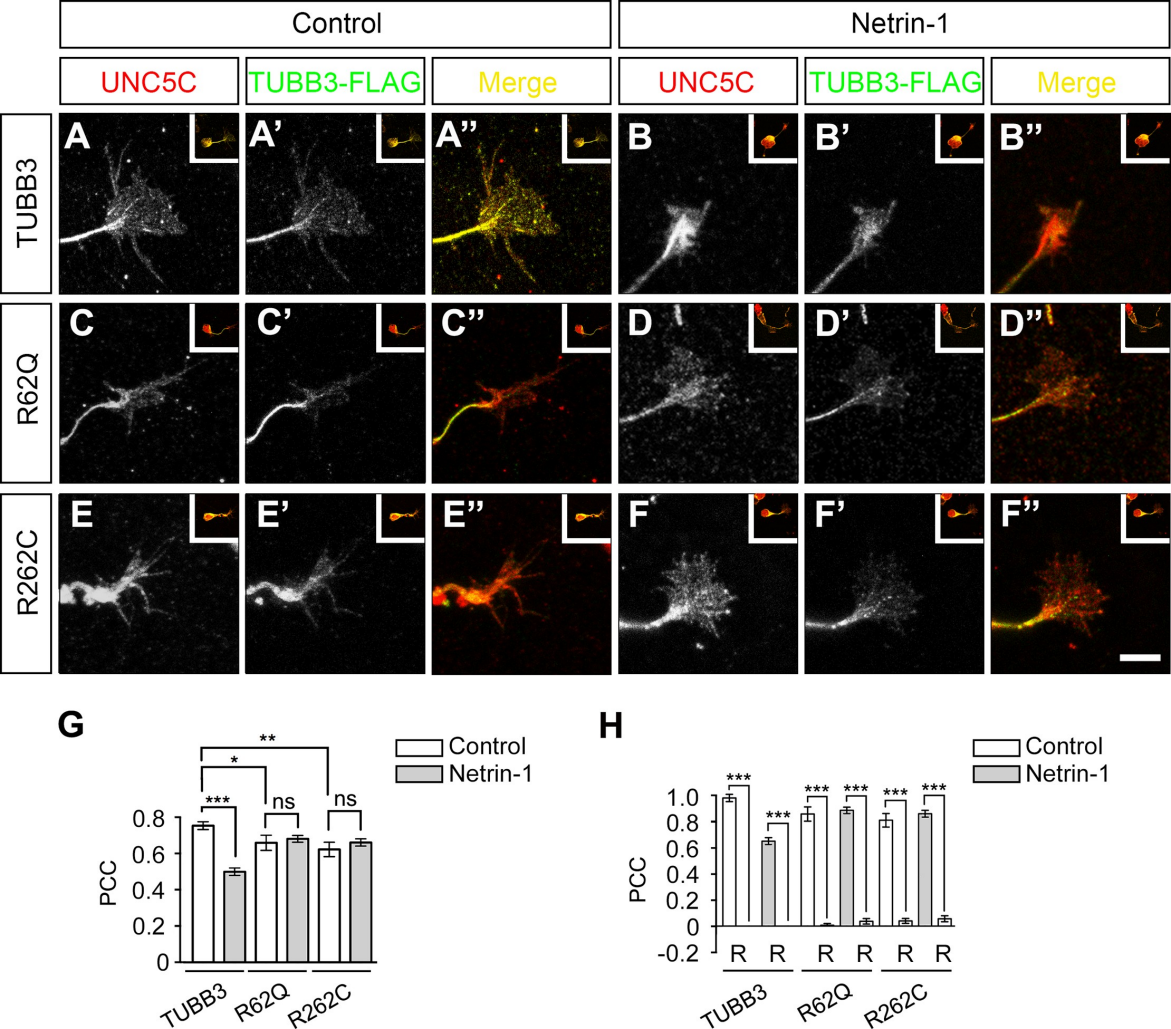
*TUBB3* mutations reduce the colocalization of UNC5C with TUBB3 in the GC of primary EGL neurons. A-F”, Primary EGL neurons from P4 mouse cerebella were co-transfected Venus-YFP with TUBB3 shRNAs plus wild-type TUBB3 (A-B”), R62Q (C-D”) and R262C (E-F”), respectively. After transfection, neurons were cultured overnight and stimulated with either sham-purified control (A-A”, C-C”, and E-E”) or purified netrin-1 (200 ng/ml, B-B”, D-D”, and F-F”). Scale bar, 10 μm. G, Quantitative analysis of PCC in the peripheral area of the GC. H, Quantification of PCC after 90 degree counterclockwise rotation of one fluorescence channel. Data are mean±SEM. ns, not significant; *, *p* < 0.05; **, *p* < 0.01; ***, *p* < 0.001 (one-way ANOVA and Tukey’s test for post hoc comparisons).

UNC5C is highly expressed in developing mouse cerebella and colocalizes with TUBB3 in the axonal GC of EGL neurons in a netrin-1 dependent manner [13, 41, 42]. To determine whether missense mutations in human *TUBB3* affect this colocalization as well as netrin-1 regulation, TUBB3 shRNAs were co-transfected with either wild-type TUBB3 or two TUBB3 mutants, R262C and R62Q, into P2 mouse EGL cells. Primary neurons were then cultured and stimulated with netrin-1. As expected [13], quantitative colocalization analysis of confocal fluorescence microscopy images showed a partial overlap of immunofluorescent signals of UNC5C and TUBB3 in the peripheral region of GCs (Fig 4A–4A", quantification in G), and netrin-1 decreased the overlap of UNC5C with TUBB3 in the GC (Fig 4B–4B", quantification in G). However, expression of either R262C or R62Q decreased the overlap of UNC5C with these TUBB3 mutants, and netrin-1 failed to regulate this overlap, compared to the wild-type TUBB3 group (Fig 4C–4F", quantification in G). To avoid artificial overlaps of random signals, we recalculated PCC value after rotating one fluorescent channel of the same confocal image by 90 degrees (Fig 4H). The PCC approximated zero in each group after rotation, suggesting a real correlation of fluorescent signals (Fig 4H) [13, 44]. These results indicate that *TUBB3* mutations disturbed the overlap of UNC5C with mutated TUBB3 proteins in the GC of primary EGL neurons and blocked the netrin-1-regulated colocalization.

### *TUBB3* mutations block netrin-1-mediated GC collapse and axonal repulsion

The GC collapse is a critical step in axon repulsion. UNC5C is required for netrin-1-induced GC collapse of P2 mouse EGL cells [39], and TUBB3 is involved in netrin-1/UNC5C-mediated axonal repulsion [13]. To determine whether *TUBB3* mutations affect the netrin-1 effect on GC collapse, we used an *in vitro* GC collapse assay of postnatal cerebellar EGL cells in the presence or absence of netrin-1. Venus-YFP plus TUBB3 shRNAs were nucleofected with wild-type human TUBB3, TUBB3 R62Q or TUBB3 R262C into cerebellar EGL neurons from P2 mouse embryos. Primary neurons were cultured and stimulated by either control or netrin-1 in bath incubation. As expected, netrin-1 increased GC collapse of EGL cells in the wild-type TUBB3 group (Fig 2A and 2B, quantification in 2G). However, netrin-1 stimulation failed to induce GC collapse in EGL neurons transfected with either R262C or R62Q (Fig 2C–2F, quantification in 2G). These data suggest that R262C and R62Q amino acid substitutions abolish netrin-1 mediated EGL GC collapse.

**Fig 5:**
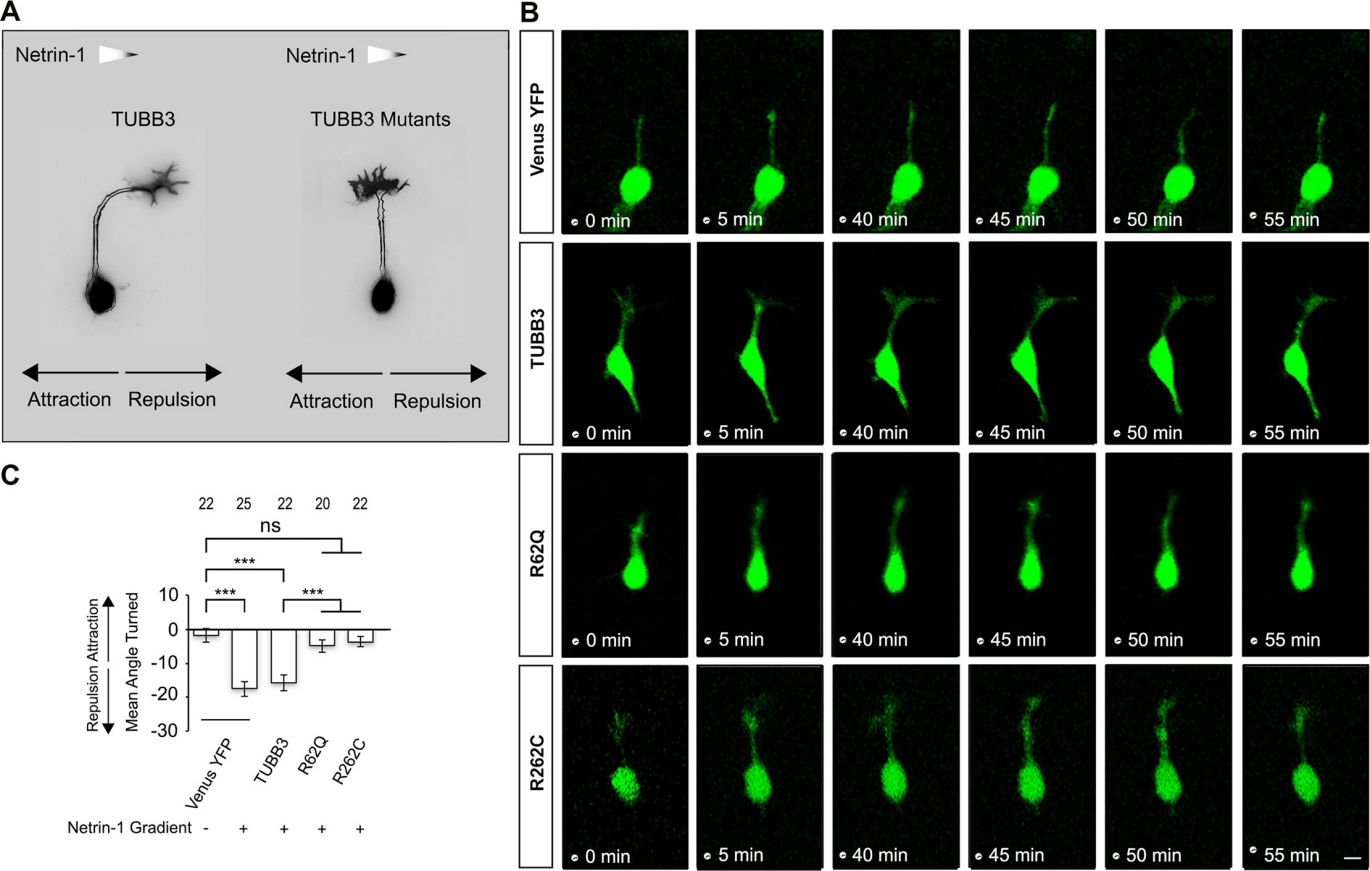
*TUBB3* mutations disrupt netrin-1-induced axon repulsion. A, Schematic diagram of axon turning of P2 mouse EGL cells co-transfected with TUBB3 shRNAs plus either wild-type TUBB3 or TUBB3 mutants in a netrin-1 gradient using a Dunn chamber axon guidance assay. B, Live-cell imaging showing axon turning of P2 EGL cells transfected with Venus-YFP only or combination of Venus-YFP, TUBB3 shRNAs plus wild-type TUBB3, R62Q, or R262C. A left-to-right netrin-1 gradient was established as shown in A. Scale bar, 10 μm. C, Quantification of axon turning of P2 EGL cells. Data are mean±SEM. ns, not significant; ***, *p* < 0.001 (one-way ANOVA and Tukey’s test for post hoc comparisons). The numbers on the top of each bar indicate the numbers of GCs analyzed in the corresponding groups.

To further examine the role of *TUBB3* mutations in netrin-1-mediated axon repulsion, either Venus YFP alone or Venus YFP plus TUBB3 shRNAs were co-transfected with wild-type TUBB3, R262C or R62Q into EGL neurons from P2 mouse cerebella, respectively, and a Dunn chamber axon guidance assay was performed (Fig 5A) [13, 40]. In this assay, the outer well of the Dunn chamber was filled with either purified netrin-1 or the sham-purified control, and GC navigation of transfected neurons immersed in the gradient of netrin-1 or control right over the bridge area was examined. The angle turned was evaluated by measuring the angle between the initial and final position of an axon. Consistent with a previous study [13], we found that netrin-1 repelled the axonal projection of EGL neurons transfected with Venus YFP alone or with the combination of TUBB3 shRNAs plus wild-type TUBB3 (Fig 5B, quantification in 5C). Expression of either R262C or R62Q in primary P2 EGL neurons abolished netrin-1-induced axon repulsion (Fig 5B, quantification in 5C). These results indicate that *TUBB3* mutations impair netrin-1/UNC5C-mediated axon repulsion.

### *TUBB3* mutations disrupt chicken DRG axon projection *in vivo*

**Fig 6:**
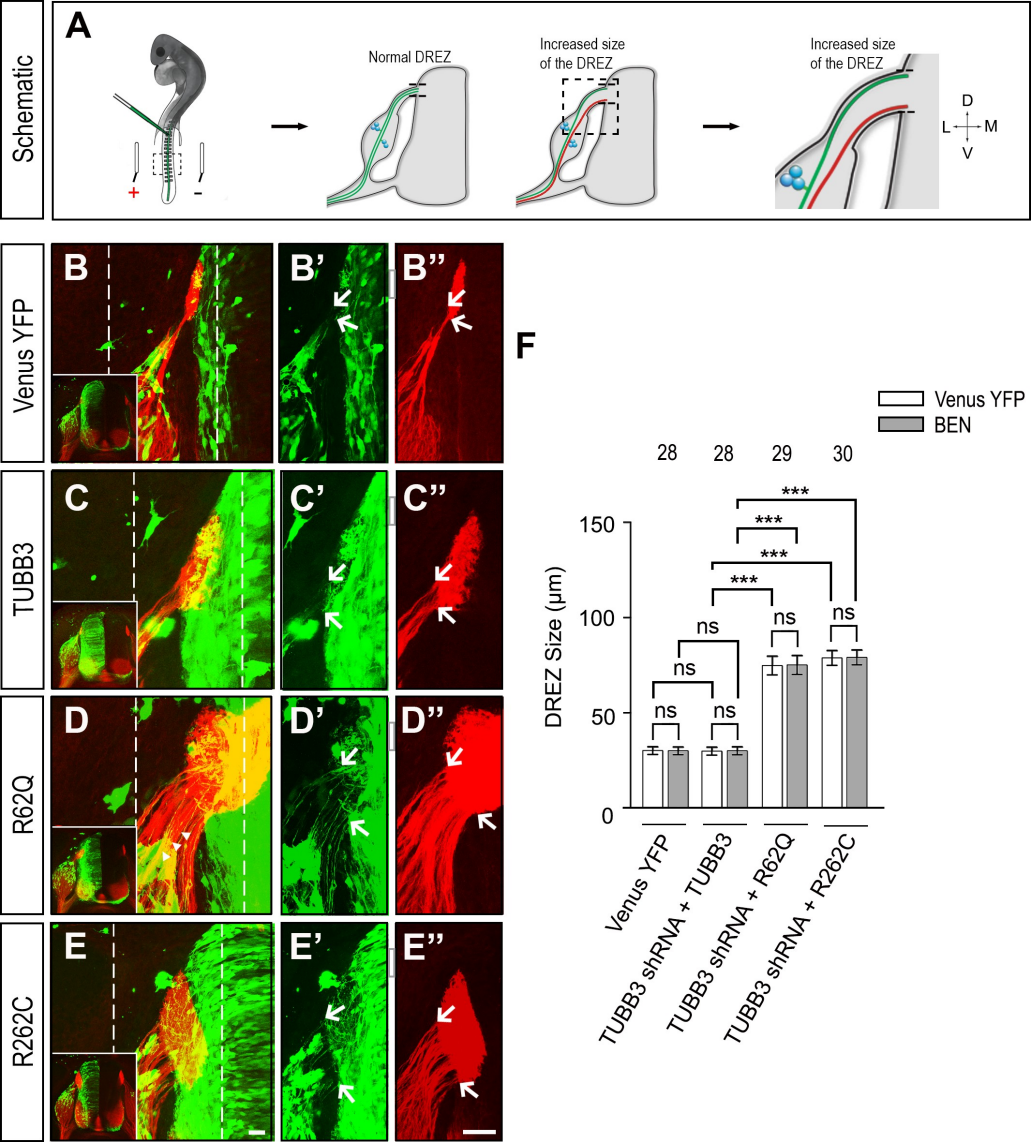
*TUBB3* mutations impair DRG axon projection *in vivo*. A, Schematic of the experimental design. The normal and defective projections of DRG axon bundles were respectively colored green and brown. B-E”, The chick neural tubes were electroporated with Venus YFP only (B-B”), Venus YFP plus TUBB3 shRNAs and wild-type TUBB3 (C-C”), Venus YFP plus TUBB3 shRNAs and R62Q (D-D”), and Venus YFP plus TUBB3 shRNAs and R262C (E-E”), respectively. Transverse sections of the spinal cords after electroporation were stained with a BEN antibody. B-E, Overlay confocal images of green (Venus YFP in B’-E’) and red (BEN staining in B”-E”) fluorescence. Corresponding low-magnification images are placed in the lower left side of B-E. B’-E’ and B”-E” are images showing axon projection of either Venus YFP- or BEN-labeled DRG neurons in the region of interest in B-E (dashed lines), respectively. Scale bar, 50 μm. White arrows point to the dorsal and ventral boundary of the DREZ. F, Quantification of the DREZ size. Data are mean ±SEM. ns, not significant; ***, *p* < 0.001 (one-way ANOVA with Tukey's test for post-hoc comparisons). The numbers on the top of each bar indicate the numbers of samples analyzed in the corresponding groups.

During development, netrin-1/UNC5C/TUBB3 signaling is involved in guiding DRG axons to project properly toward the dorsal root entry zone (DREZ) and preventing them from entering aberrantly into the spinal cord [13, 45–48]. To further examine the role of *TUBB3* mutations in DRG axonal projection, either Venus YFP alone or Venus YFP plus TUBB3 shRNAs were electroporated with wild-type TUBB3, R262C or R62Q into the chicken neural tube at HH stage 16 [13, 46]. YFP-labeled embryos at HH stages 23–25 were collected, and transverse sections of the lumbosacral segments of the spinal cords were immunostained with axonal-specific BEN antibodies as described in previous studies (Fig 6A) [13, 46]. As expected, we found that DRG axons projected normally toward and entered the dorsal spinal cord at the DREZ in embryos expressing Venus YFP only (Fig 6B–6B'', quantification in 6F). Expression of TUBB3 shRNAs plus wild-type TUBB3 didn't affect DRG axon projection toward the spinal cord, compared to the Venus YFP group (Fig 6C–6C'', quantification in 6F). However, expression of TUBB3 shRNAs plus either R262C or R62Q dramatically increased DREZ size (Fig 6D–6E'', quantification in 6F). These data suggest that *TUBB3* mutations affect netrin-1/UNC5C-mediated DRG axon projection and pathfinding in the developing spinal cord.

## Discussion

**Fig 7:**
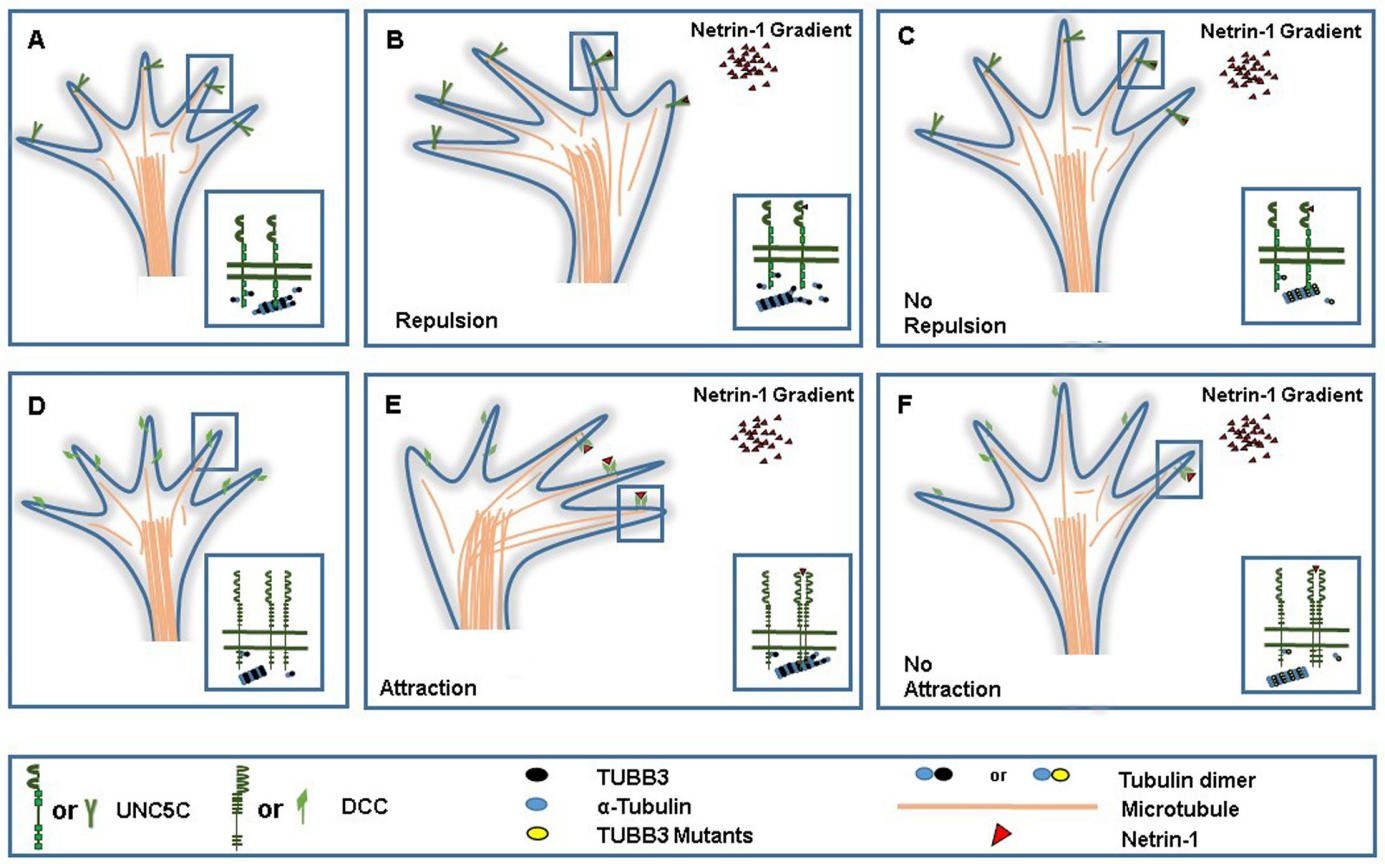
*TUBB3* mutations respectively disrupt netrin-1/UNC5C-mediated axon repulsion and netrin-1/DCC-mediated attraction. A-C, A schematic model of the role of *TUBB3* mutations in netrin-1/UNC5C repulsive signaling. In the absence of netrin-1, UNC5C interacts with unpolymerized and polymerized TUBB3 in microtubules in the peripheral region of the GC (A). In the presence of a netrin-1 gradient, netrin-1 binding to UNC5C increases microtubule polymerization in the GC and disproportionately dissociates the interaction of UNC5C with polymerized TUBB3 in MTs on the side of the GC close to the netrin-1 gradient, differentially triggering GC collapse on that side and axon repulsion from the netrin-1 gradient (B). Mutations in *TUBB3* impede the netrin-1-mediated dissociation of UNC5C with polymerized TUBB3 in microtubules, impairing netrin-1-mediated GC collapse and axon repulsion, whereas TUBB3 mutants still incorporate into microtubules and netrin-1 increases microtubule polymerization in the GC. D-F, A working model of the role of *TUBB3* mutations in netrin-1/DCC attractive signaling. DCC, unpolymerized TUBB3, and polymerized microtubules are presented in the peripheral region of an axonal GC in the absence of netrin-1 (D). Netrin-1 increases DCC homodimerization, microtubule polymerization, and interaction of DCC with polymerized TUBB3 in microtubules on the side of the GC close to the netrin-1 gradient, which polarizes the dynamic MTs to project towards the netrin-1 gradient, resulting in axon attraction (E). Mutations in *TUBB3* disrupt the netrin-1-mediated coupling of DCC to polymerized TUBB3 in microtubules in the GC, blocking netrin-1-mediated axon attraction.

During brain development, modulation of microtubule dynamics in the GCs of developing neurons is essential for proper axon elongation, branching, and projection. Mutations in the *tubulin* genes perturb microtubule dynamics and result in a broad spectrum of brain malformations associated with axon guidance and neuronal migration defects [6, 14]. However, the molecular mechanism underlying these neurodevelopmental disorders are not well understood. Direct coupling of netrin receptors DCC, DSCAM, and UNC5C to microtubules via dynamic TUBB3 plays a key role in netrin-1-induced axon outgrowth, branching, and guidance (Fig 7)[13, 23, 25]. A recent study has shown that missense mutations in the *TUBB3* gene disrupt the engagement of netrin/DCC signaling with microtubule dynamics, resulting in specific defects in netrin-1-mediated axon attraction (Fig 7) [24]. The results here demonstrate that disease-associated missense mutations in the human *TUBB3* gene could impair the coupling of netrin-1/UNC5C signaling with dynamic TUBB3 in microtubules as well as netrin-1-mediated axon repulsion in the developing nervous system (Fig 7).

TUBB3 can interact with UNC5C *in vitro* and netrin-1 reduces this interaction in primary neurons [13]. To determine the effects of disease-related missense mutations in *TUBB3* on the binding to UNC5C, we performed co-IP experiments after co-transfection of UNC5C with either wild-type TUBB3 or a single TUBB3 mutant into HeLa cells (Fig 1). We found that UNC5C interacts with wild-type TUBB3 and most of TUBB3 missense mutants (Fig 1). However, the interaction of UNC5C with five out of twelve TUBB3 mutants (A302T, M323V, R262C, R62Q, and D417H) was significantly reduced compared to the wild-type TUBB3 (Fig 1), suggesting that *TUBB3* mutations may differentially perturb the interaction with UNC5C. Similarly, endogenous UNC5C also interacted with wild-type TUBB3 and T178M, but the interaction with R262C and R62Q, the most and least frequent mutant substitutions in the *TUBB3* gene, was significantly reduced in primary neurons (Fig 1D). Netrin-1 increases microtubule dynamics in the GC of cerebellar EGL neurons, but reduces the interaction of UNC5C with polymerized TUBB3 in microtubules (Fig 3) [13]. Expression of either R262C or R62Q did not affect the netrin-1-promoted polymerization of these mutants into microtubules (Fig 3), however, it blocked netrin-1-regulated cosedimentation of UNC5C with stabilized microtubules (Fig 3). To further reveal whether *TUBB3* mutations affect netrin-1-dependent regulation on these interactions, we employed a colocalization analysis of endogenous UNC5C with TUBB3 mutants in the GC of developing cerebellar EGL neurons using confocal fluorescence microscopy (Fig 4). UNC5C partially colocalized with TUBB3 in the P region of the GC and netrin-1 reduced this colocalization (Fig 4A–4B”, 4G and 4H) [13]. However, expression of either R262C or R62Q inhibited the colocalization of UNC5C with these mutants and netrin-1 failed to regulate this colocalization (Fig 4C–4H). Altogether, these results suggest that *TUBB3* mutations could disrupt the netrin-1-regulated interaction of UNC5C with polymerized TUBB3 in microtubules in the developing neurons.

TUBB3 is a highly dynamic β–tubulin isoform in neurons, which contains an N-terminal domain, an intermediate region, and a C-terminal domain. Five of twelve *TUBB3* missense mutations impaired the interaction with UNC5C are widely distributed in these domains, suggesting that *TUBB3* mutations may cause conformational changes of TUBB3 protein, disrupting the interaction with UNC5C. UNC5C binds to both unpolymerized and polymerized TUBB3 and the netrin-1-reduced interaction of UNC5C with polymerized TUBB3 in microtubules is required for netrin repulsion [13]. Both R62Q and R262C disrupt the netrin-1-regulated interaction of UNC5C with polymerized TUBB3 (Fig 3). Residue R62 is located in a region associated with lateral interactions of microtubule protofilaments, which regulates microtubule dynamics [17, 49]. Residue R262 mediates interactions with motor and non-motor microtubule-associated proteins and contributes to regulation of microtubule polymerization dynamics at the plus- and minus-ends [44, 50]. Missense mutations in residue R262 can alter microtubule dynamic instability by increasing the stability of microtubules at both ends [50]. Therefore, it is plausible to propose that missense mutations in R62 and R262 may change microtubule dynamics and impair netrin-1-regulated interaction of UNC5C with polymerized TUBB3 in microtubules (Fig 7). Interestingly, UNC5C failed to interact with R262C, but not R262H (Fig 1A and 1B). Previous studies have shown that different missense mutations at the same position of TUBB3 such as A302V/A302T or R262C/R262H could cause contrasting phenotypes [16, 51]. Although the mechanisms responsible for these different effects remain unknown, a substitution from a positively charged amino acid (R or H) to polar neutral amino acid (C) could change TUBB3 structure, which affects microtubule dynamics and alters the interactions with other effector proteins such as UNC5C, resulting in profoundly different outcomes [16, 51]. Further study is needed to gain insight into the roles of specific *TUBB3* mutations in netrin-1/UNC5C-mediated microtubule dynamics in the GC of primary neurons.

Missense mutations in the human *TUBB3* gene disrupt microtubule dynamics and kinesin interactions, resulting in specific defects in axon guidance and neuronal migration [16, 17]. Given that TUBB3 is widely expressed in all developing neurons, how these mutations cause guidance defects in a spatio-temporal manner in the developing nervous system remains unclear. A recent study shows that *TUBB3* mutations specifically impair netrin-1/DCC-mediated axon outgrowth, branching, and attraction [24]. To further determine whether *TUBB3* mutations affect netrin-1-promoted axon repulsion, mouse EGL neurons were transfected with wild-type TUBB3, R62Q or R262C and the functional roles of these proteins were studied by both *in vitro* and *in vivo* experiments. Netrin-1 promoted both GC collapse and axon repulsion of EGL cells expressing wild-type TUBB3 (Figs 2 and 5). In contrast, netrin-1 stimulation failed to induce GC collapse and axon repulsion of EGL neurons transfected with either R62Q or R262C (Figs 2 and 5). In addition, results from *in ovo* electroporation studies with chick spinal cords indicate that expression of either R62Q or R262C, but not wild-type TUBB3, disrupts DRG axon projection toward the dorsal spinal cord (Fig 6). Altogether, these findings suggest that missense *TUBB3* mutations perturb netrin-1-mediated GC collapse and axon repulsion during development.

Previous studies have shown that UNC5 collaborates with DCC or DSCAM to promote axon repulsion [39, 52, 53] and the interaction of polymerized TUBB3 in microtubules with DCC and DSCAM or UNC5C respectively mediates netrin-1-induced axon attraction or repulsion [13, 23, 25]. It remains to be determined whether *TUBB3* mutations impair the interaction with DSCAM as well as the coordination of DCC, DSCAM, and UNC5C on modulation of microtubule dynamics in netrin repulsive signaling. Both UNC5A and UNC5B, two homologues of mammalian UNC5 are involved in netrin repulsive signaling [42, 54]. As most of *TUBB3* mutations (7 out of 12) do not affect the interaction with UNC5C (Fig 1), it will be interesting to further characterize whether these UNC5 homologues can interact with TUBB3 and missense mutations in the human *TUBB3* gene perturb these interactions, resulting in axon guidance defects in netrin-1-mediated repulsion. *TUBB3* mutations are associated with a wide range of neurodevelopmental disorders, mainly reflecting defects in axon projection and neuronal migration [16, 17]. Although most of TUBB3 mutants revealed a significant reduction of DCC and UNC5C binding, several disease-associated substitutions E205K, M388V, and E410K could still interact with both DCC and UNC5C (Fig 1) [24], suggesting other guidance signaling besides the netrin/DCC/UNC5C/TUBB3 pathway may also be affected. It is well known that GC navigation of developing neurons is controlled by a variety of extracellular signals including guidance cues, growth factors, and cell adhesion molecules, such as ephrins, Semaphorins, reelin, Sonic hedgehog, Wnts, slits, and bone morphogenetic proteins [2, 7, 55–59]. Further studies are necessary to determine whether disease-related *TUBB3* mutations affect signaling mechanisms downstream of these guidance cues.

## Supporting information

S1 Fig Three biological replicates of co-IP experiments related to Fig 1A. Distinct HeLa cell samples were collected from three independent transfection and co-IP experiments were performed as described in Fig 1A.
(TIF)

S1 Data A collection of quantitative data related to Figs 1–6. (XLSX)

## Abbreviations

AC: Anterior Commissure
CC: Corpus Callosum
DCC: Deleted in Colorectal Cancer
DREZ: Dorsal Root Entry Zone
DRG: Dorsal Root Ganglion
DSCAM: Down Syndrome Cell Adhesion Molecule
EGL: External Granule Layer
GC: Growth Cone
HH: Hamburger and Hamilton
MCD: Malformations of Cortical Development
PAGE: Polyacrylamide Gel Electrophoresis
PCC: Pearson’s Correlation Coefficient
PLL: Poly-L-Lysine
TUBB3: Tubulin Beta-III
UNC5: Uncoordinated-5
UTR: Untranslated Region
