# Supplementary figures and images for: Disease-associated mutations in human TUBB3 disturb netrin repulsive signaling
Source: PLoS One. 2019 Jun 21;14(6):e0218811. doi: 10.1371/journal.pone.0218811 (PMC6588280; doi:10.1371/journal.pone.0218811)

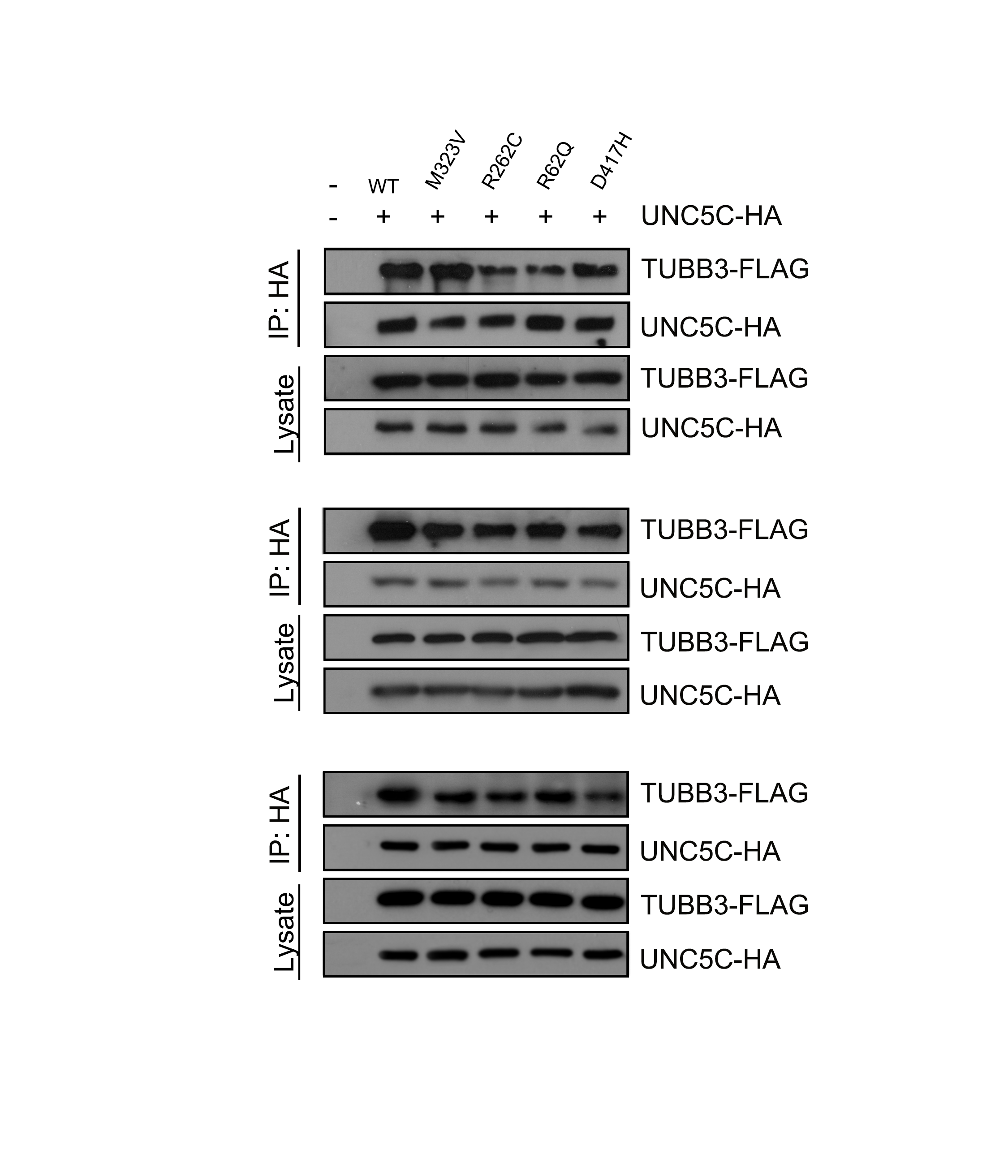

Supplement: S1 Fig — Distinct HeLa cell samples were collected from three independent transfection and co-IP experiments were performed as described in Fig 1A. (TIF) [file pone.0218811.s001.tif]
